# Verification of the Mismatch Negativity (MMN) responses in normal adult subjects

**DOI:** 10.1016/S1808-8694(15)31176-9

**Published:** 2015-10-19

**Authors:** Adriana Bortoleto Brossi, Karen Cristina Borba, Cristiane Fregonesi Dutra Garcia, Ana Cláudia Mirândola Barbosa Reis, Myriam de Lima Isaac

**Affiliations:** 1 Speech and Hearing Therapist; 2 Speech and Hearing Therapist; 3 M.S in Public Health - USP; PhD - Department of Otorhinolaryngology, Ophthalmology, Head and Neck Surgery - Medical School of Ribeirão Preto/SP-USP, Professor of Speech and Hearing Therapy - Franca - Unifran; 4 PhD in Human Communication Disorders - UNIFESP/EPM, Professor of Speech and Hearing Therapy - Universidade de Franca - Unifran; 5 PhD of Pediatrics - Medical School of Ribeirão Preto/SP - University of São Paulo, USP., ENT. Professor of Otorhinolaryngology - Department of Otorhinolaryngology, Ophthalmology and Head and Neck Surgery - Ribeirão Preto Medical School/SP -USP; Curso de Fonoaudiologia - Universidade de Franca Departamento de Oftalmologia, Otorrinolaringologia e Cirurgia de Cabeça e Pescoço da Faculdade de Medicina de Ribeirão Preto/SP - USP

**Keywords:** auditory cortex, evoked auditory potentials, event related potentials

## Abstract

Mismatch Negativity (MMN) is used to evaluate the central auditory system.

**Aim:**

to characterize the MMN, in normal subjects.

**Materials and Methods:**

prospective study, 12 subjects, six males and six females, between the ages of 18 and 24. “Mann-Whytnei” test. **Exams**: Pure Tone Audiometry (PTA), Tympanometry, Otoacoustic Emissions and Short and Long Latency Auditory Potentials (MMN).

**Results:**

in MMN variable amplitude, the mean value was of −2.757 μV and −3.548 μV, CZA1 and CZA2; of 1.435 μV and −1.867 μV, CZA1 and CZA2. In variable and medium latency, we found in 150.7ms and 153.2ms, CZA1 and CZA2; in 170.4ms and 184.0ms, CZA1 and CZA2 - for females and males respectively.

**Conclusion:**

related to latency, there was significant statistical difference between the genders in relation to CZA1 and CZA2; and it was lower for females and higher for males.

## INTRODUCTION

Hearing is the means through which the individual is able to acquire information. It allows language acquisition and development and, consequently, fosters school learning. Hearing encompasses a peripheral and a central part, and it is necessary to have both systems working together because learning is directly associated with these factors.

This function can be impaired in two ways, peripheral hearing loss and auditory processing disorder, and the later is associated with the detection and interpretation of sound stimuli. If there is any alteration in the sound stimulus for the central nervous system, the information received will be distorted and will make it difficult to process and interpret sound^1^.

Peripheral hearing assessment should include basic procedures such as Tonal Audiometry (TA), Rate of Speech Recognition (RSR), Acoustic Immitance, and other tests related with auditory processing.

The Brainstem Evoked Auditory Potential (BEAP), also known as short latency Auditory Evoked Potential (AEP), provide information about the auditory nuclei which are located in the brainstem, by means of waves generated up to 10 milliseconds (ms) after auditory stimulus, and aims at confirming or ruling out any brainstem involvement. In middle latency AEP, the waves happen between 10 and 80ms after stimulus presentation, they involve the thalamus-cortical pathway, mesencephalic reticular formation and the inferior colliculus.

Long latency AEP capture auditory responses above 80ms, reflecting cortex and thalamus activity^2^.

Long latency AEP are classified in exogenous and endogenous potentials. The former depend on stimulus characteristics (intensity, frequency, duration) and the later is influenced by cognitive skills (Sutton et al. (1965 apud 3). The endogenous potentials are also called cognitive potentials or are associated with events or tasks. They are recorded as Mismatch Negativity (MMN), with latency of 150 to 275ms and P300, with latency of 220 to 380ms (Kraus; McGree,1996 apud 3).

MMN points indicate a difference in response between a rare stimulus together with a frequent stimulus and, contrary to P300, does not require patient attention.

MMN is present early on and reaches its peak values in the early school age (Kraus; McGree, 1994 apud 4).

Clinical audiology has become a broad research field, especially with the use of objective assessment procedures for the auditory system. Parallel to that, technological progress has brought about a survival for risk groups (such as premature children, those with syndromes and others) and they may develop alterations in the Central Auditory System (CAS) throughout their existences.

The MMN test is indicated to assess CAS responses, we chose to check them in a healthy population in order to characterize them so as to include this test in audiologic practices, aiming at effectively establishing the differential diagnosis.

Therefore, the present investigation aims at characterizing MMN responses in normal adult subjects of both genders with ages between 18 and 25 years.

### Mismatch Negativity (MMN)

One of the traits of these long latency potentials is that they are not affected by the physical properties of the stimulus, because the functional use of the stimulus by the individual has an impact on its evocation, in other words, the response may be determined more due to paying attention to the stimulus then by its frequency or intensity^4^.

Under normal conditions, a series of identical repeated stimuli cause a negative wave around 100ms after the stimulus (N1). If a different stimulus is inserted within the series of stimuli, N1 will still happen and there will be an additional negative peak that will remain for 100ms more. This other peak is called Mismatch Negativity or MMN^5^.

**Figure 1:**
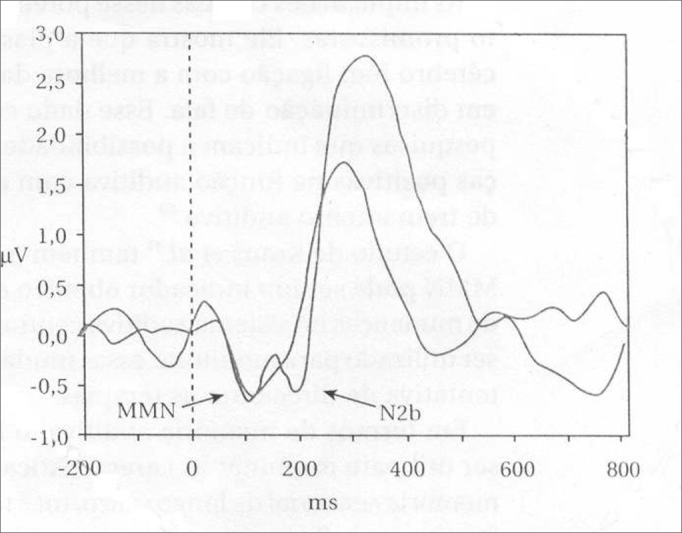
Long latency auditory evoked potential diagram - MMN - Source: SCHOCHAT, 2004, p. 665.

Figure 1 shows the MMN trace.

Long latency AEP are characterized by an initial peak between 60 and 80ms (P60) with an amplitude of approximately 7μV and a 15ms width; the second appears between 90 and 100ms (N100) and it is negative, with an approximate amplitude of 10μV and a width of 40 to 50ms; the third is around 100 and 160ms (P160), positive with an approximate amplitude of 6μV and 40 to 50ms of width; the fourth happens between 180 and 200ms (N200); negative, with an amplitude of 6μV and 70ms of width. At each scan, two waves are traced, one for the constant stimulus and another for the rare stimulus. One wave is subtracted from the other (rare is shorter than the constant) and the result is a negative wave^4^.

The recommended setup for MMN is usually more central, using positions Cz, Fz or Pz. The filter to be used to record this potential should be between 1 and 100 Hertz (Hz). The window to be used must have 750ms, at a speed of 1.1 stimulus per second. We are still unsure about how many stimuli should be averaged; notwithstanding, it is believed that 10 to 15% of them must be rare, and the rest of them must be frequent4.

MMN is an automatic response of attention prior to the change in stimulus, being a potential clinical instrument for the objective assessment of patients who have difficulties in communicating or who are being investigated as to their hearing. One of the most relevant applications of this potential could be the detection of articulation disorders, for example when children mispronounce the |r| or the |l|, it may be difficult to ascertain if this is an articulation or hearing problem in which the child has difficulties in correctly understanding the phoneme. MMN can provide information about the physiological bases for hearing discrimination, without the need for the child to speak^4^.

The clinical applications of this potential show that brain plasticity has connections with an improvement in speech discrimination. In relation to auditory memory, MMN could be useful in order to examine the characteristics of not only the long term sensorial memory, but also of the factors that impact the adding of sensorial information that remains stored in memory for a long time^4^.

MMN seems to reflect the neuronal representation of discrimination of numerous auditory aspects. If this response reflects the skill to discriminate acoustic stimuli it would have clinical value, because speech perception depends on a neural response to a change in stimulus. MMN is triggered by one frequent stimulus and a rare one; however, contrary to P300, attention is not required, in other words, the patient shall remain seated and relaxed and, if possible, watching a video (without sound) for distraction purposes and not pay attention to the sound stimulus that is presented to him^4^.

MMN can become a valuable tool to investigate sound discrimination and sensorial memory listening skills in children; however, we still need more research on recording patterns, patient status and age-related normative data in order to use it as a method for clinical evaluation.^3^

In order to trigger MMN, a standard 698 Hz stimulus and a deviant stimulus which varied between 12 and 99 Hz were presented in different acquisitions. Interestimulus interval variation during MMN acquisition provide a discrimination and auditory sensorial memory evaluation^3^.

Elias, Peixoto and Mendonça, 20046 reviewed the literature on the MMN test, with a publication from 2003 and the first semester of 2004, and found 12 international papers on the subject.

Following, we will present a summary of the papers aforementioned in a chronologic and alphabetic order and we shall include the papers published in the second semester of 2004 and in the first semester of 2005.

Kujala et al. (2003)^7^ analyzed seven subjects with ages between 19-23 years on the neuroplastic changes that happen in cortical memory. They suggested that there is a close association between neural representation of sound patterns and phonemes.

Martin et al. (2003)8 observed 55 children and 12 adults with ages between 4-11 and 22-38 years, respectively. They concluded that the MMN generation or its orientation in neurological processes cover the discrimination of simple sounds and at 11 years of age they are still immature.

Shinozaki et al. (2003)^9^ studied ten subjects with ages varying between 25-43 years. They suggested that there is a bidimensional integration with important restrictions to the neurological processing of the acoustic environment present in the human brain.

Shtyrov, Hauk and Pulvermüller (2003)10 studied thirty subjects between 18-40 years. They indicated that the activation of neurons which work as memory traces, involves cortical sensory-motor structures in order to code words.

Sittiprapaporn et al. (2003)^11^ studied nine subjects with ages varying between 18-35 years. They concluded that the MMN responses are higher when triggered by sounds from native tongue speech; in the left auditory cortex there is a higher contribution to speech; MMN reflects the presence of long term memory in the recognition of words.

Uther et al. (2003)^12^ analyzed ten subjects with ages varying between 18-25 years and suggested that MMN and MMNm are related to the auditory temporal resolution, representing measures which do not depend on attention.

Winkler et al. (2003)^13^ studied three subjects with ages varying between 18-25 years, suggesting that the information stored in the sensorial memory can be accessed by processes operated in the auditory cortex.

Endrass, Mohr and Pulvermüller (2004)^14^ studied 17 subjects with ages varying between 24-27 years and observed that the MMN test is associated with the word, because it allows the summation of neurological activities between the brain hemispheres.

Giaquinto (2004)15 reported that evoked potentials (EPs) are associated with extreme stimuli and appear after a short interval and the event-related potentials (ERP) are associated with cognitive processes, which involve attention, discrimination and the execution of tasks. Both may provide prognostic clues in individuals with acute brain lesions, in other words, accuracy in the rehabilitation of these individuals. The author suggests that the absolute values should not be used for prognosis because of variations in long latency AEP.

Grimm, Widmann and Shroger (2004)16 studied 12 subjects with ages varying between 19-35 years. They concluded that the duration of the stimulus presented depends on the attention span and focus. For long duration sounds, when there is attention, processing is prevented.

Kanoh, Futami and Hoshimiya (2004)^17^ observed that MMN is a potential that reveals the sensorial memory process. The auditory stimulus that consists on a series of exposure of tones showed that MMN collects an alteration in the frequency of the last tone in the stimulus and that its intensities change according to the number of tones and the frequency of the sequence to be memorized.

Lonka et al. (2004)^18^ studied five subjects with ages between 28-55 years. They noticed a better MMN capture in cases of hearing loss and related such fact to the cortical reactivation of memory for phonemes, because it is associated with the cortical plasticity related to auditory memory and the discrimination re-learned in the processing of speech sounds after cochlear implant.

Näätänen et al. (2004)^19^ studied seven subjects with 25 years of age. They proposed a new model that can provide five different MMN at the same time, but only one MMN is obtained. This new model will establish the different skills associated with hearing discrimination in a short recording time.

Pettigrew et al. (2004)^20^ studied MMN responses in a variety of stimuli from discourses in a multiple attention paradigm, with a contrast of fine discourse. They concluded that MMN responses can be obtained by means of discourse stimuli with separate acoustic alterations within the sketches of a multiple altered paradigm, in other words, it has positive clinical implications for the tests with diseased patients.

Shafer, Shawartz and Kaurtzberg (2004)^21^ studied 42 subjects with ages between 28 and 30 years. Their results indicated that Hindu speakers used detailed cerebral acoustic-phonetic information faster than their English counterparts.

Zeftawi (2004)^22^ reported that the primary and non-primary auditory pathway contribute in MMN generation; the non-primary pathway contribute to the process of durational changes, while the primary pathway contributed to the process of spectro-temporal changes. In the present investigation we found statistically significant differences between durational and spectro-temporal contrasts by latency and duration. This was attributed to acoustic and physiological differences between the auditory pathways. It can be used in intensive analysis of patients with auditory neuropathies and aphasia, and to help differentiate cortical and subcortical lesions that affect speech processing.

Carrl, Corral, Escera (2005)^23^ tested the potentials associated with events in healthy individuals in order to test the human brain's accuracy, aiming at collecting abstractive auditory rules, which were determined by the relationship of frequency between two pure tones that formed a pair. The pairs had the frequencies of identical tones and the altered pairs had in the second, two tones, four, six and eight higher or lower musical levels. All abstractive changes attained MMN.

Molholm et al. (2005)^24^ reported that the neural generators of the change detection auditory system vary in function of the characteristics of the stimuli obtained: repetitive tones in differential activation patterns related to MMN in the auditory cortex. MMN indicates not only that a change happened, but also the nature of the change. This study supports a MMN model in which the sensorial memory is a specific characteristic associated with the acoustic regularities in the environment.

Näätänen, Jacobsen and Winkler (2005)^25^ reported that the MMN is an electromagnetic response for any discernible change in regular auditory intensity. This response is interpreted by an automatic cortical change detection process, in which the difference is found between the current intensity and the representation of the regular aspects of the present auditory intensity.

Rosburg et al. (2005)^26^ reported that MMN is obtained by means of auditory stimulation changes and reflects a pre-attention mechanism. The auditory evoked potentials were recorded in an intracranial way and sensitive contacts of electrodes for stimulus alteration. They were selected in order to unveil the participation of different cerebral areas for MMN production. Most electrodes with an MMN signal were located near the superior temporal lobule.

Schirmer, Striano and Friederici (2005)^27^ researched about the emotional tone of voice in men and women and observed that, regardless of gender, MMN appeared as an event-related potential, an indicator of pre-attention acoustic change detection.

Sussman (2005)^28^ analyzed the auditory aspect by means of a sound change detection sequence (MMN). He showed that the segregation processes can occur without attention focused in the sounds and how the sound elements are integrated and represented in the auditory memory. The model of the MMN results showed that the integration of sound elements with a set of sound that occur after sound segregation in independent courses, and suggests that the auditory aspect is quickly organized in distinctive courses and the integration of sequential elements for perceptual units is carried out in distinctive pre-formed courses and provides some flexibility to identify changes in sound models in the appreciation of music or speech understanding.

Winkler et al. (2005)^29^ studied the role of attention in the auditory and visual modes. They used MMN and vMMN potentials, respectively. The sequences were of two stimuli that happened frequently, which differentiated from one another in two characteristic stimuli (model stimulus) and two infrequent stimuli (altered). They concluded that responses from altered stimuli of similar patterns crossed the different attention conditions. Such results suggest that the memory representations involved in the MMN detection responses decoded the combinations of characteristics that happened frequently, and the tests sequences are assisted or not.

## MATERIAILS AND METHODS

This work consisted of a deductive research line, a descriptive, observational, cross-sectional, static, comparative, prospective study. Research focused on diagnosis.

**Figure 2:**
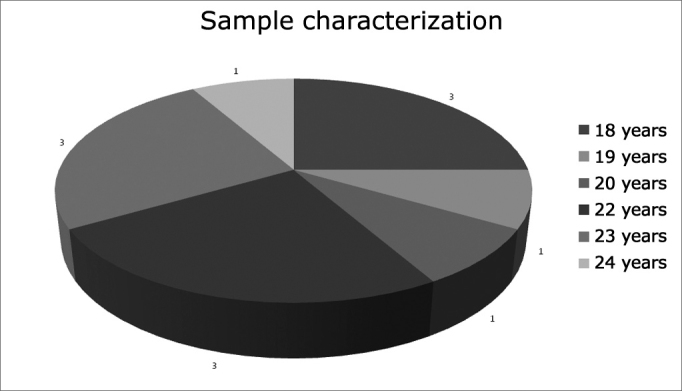
Study sample characterization - legend next to the graph

The study group had healthy adult subjects of both genders. The sample, had six males and six females, with ages ranging between 18 and 24 years (Table 1 and Figure 2).

**Table 1:** Sample subjects classified according to gender and age (N = 12)

Subject	Gender	Age (a/m)
1	F	20a 5m
2	F	22 a
3	F	22a 11m
4	F	23a 11m
5	F	23a 11m
6	F	24a 11m
7	M	18a 2m
8	M	18a 4m
9	M	18a 9m
10	M	19a 2m
11	M	22a 11m
12	M	23 a

Legend: F = female

a = years

M = male

m = months

In the analysis of results, we included the subjects with normal hearing thresholds, proper middle ear conditions for the exams to be held, otoacoustic emissions results and PEATE within normal limits.

The dependent variables studied were the results of MMN tests and the independent variables were the CZA_1_ (left side), CZA_2_ (right side) derivatives and genders (male and female).

The materials used were: sulphite, pen, pencil, eraser, cotton, gauze, probes of different sizes, cones, micropore, abrasive paste, steel wool, spatula, scissors, authorization protocols to participate in the study, data collection protocols.

The equipment used was: HEINE mini 2000 otoscope; audiometer (AC 33, phone TDH 39), calibrated on 04.18.2005; immitancemeter (AZ − 7), calibrated on 04.18.2005; Otoacoustic Emissions Analyzer (ILO 292, version 5.0, Otodynamics LTDA); Biologic version 5.70, model 317, two channels; regular computer.

The data collection procedure happened in the following stages:

1st stage: Assessment by the Ethics Committee, being approved under protocol # 037/05.

2nd stage: Signed informed consent by the participants.

3rd stage: Individual interview with the subjects in order to collect data about hearing and general health status.

4th stage: External acoustic meatus exam, in order to check its condition and then, perform the specific exams.

5th stage: Tonal audiometry and tympanometry in order to obtain tonal thresholds and middle ear conditions, normality in this exam was required for both tests. OAE and PEATE exams and the subjects needed to have TOAE present and PEATE with adequate morphology, absolute latency and interpeak within normal ranges (Table 2).

**Table 2:** Results considered pre-requisites for including the subjects in the study (N = 12).

ATL	TIMP	EOAT	ABR				
X = OD (dBHL)	X = OE	OD	OE	OD	OE	OD	OE
10	5	A	A	Pres	Pres	Ad	Ad
5	0	A	A	Pres	Pres	Ad	Ad
5	5	A	A	Pres	Pres	Ad	Ad
10	5	A	A	Pres	Pres	Ad	Ad
10	5	A	A	Pres	Pres	Ad	Ad
5	10	A	A	Pres	Pres	NR	NR
5	5	A	A	Pres	Pres	Ad	Ad
10	10	A	A	Pres	Pres	NR	NR
5	10	A	A	Pres	Pres	NR	NR
0	5	A	A	Pres	Pres	Ad	Ad
5	0	A	A	Pres	Pres	Ad	Ad
5	10	A	A	Pres	Pres	Ad	Ad

ATL - Tonal Threshold Audiometry; X - Mean Value; OD - Right Ear; OE - Left Ear; dBHL - decibel hearing level; TIMP - Tympanometry; A - Type A tympanometry; EOAT - Otoacoustic Emission; PRES - present; ABR - Auditory Brainstem Response; NR - Not done (too much noise); Ad - Adequate (absolute and relative latency within normal limits, favorable amplitude).

6th stage: MMN with pure tone. The test parameters were based on references 3 and 4 and can be seen on Table 3.

**Table 3:** Parameters used for the MMN test application.

		Tone burst
		1000 Hz (frequent)
	Frequency	With 80% (400 stimuli)
Stimulus		2000 Hz (rare)
		With 20% (100 stimuli)
	Intensity	75 dBHL
		(frequent and rare)
		Active - vertex (CZ)
Acquisition	Electrodes	Reference - left ear (A_1_)
		Right ear (A_2_)
		Ground - forehead (FPZ)
Patient	Status/ task	Awake without attention
		Watch a movie
Analysis	Replication	Difference between rare and frequent stimulus in derivations CZA_1_ e CZA_2_

In order to compare genders as to latency and amplitude measures, we chose the Mann-Whitney 30 statistics, because we had continuous values, measured in an interval scale, coming from independent groups and there was no prior consideration about the populational distribution of the variables. The level of significance (p value) was fixed in 5% (0.05). Results will be presented in the following chapter, in absolute values by means of tables.

## RESULTS

Table 4 shows the values for MMN tests obtained through the sample in the present study.

**Table 4:** Description of latency and amplitude values according to derivations CZA1 and CZA2 of the subjects in the study.

	Derivation CZ/A_1_		Derivation CZ/A_2_	
	Latency (ms)	Amplitude (μV)	Latency (ms)	Amplitude (μV)
1	152.20	− 2.45	153.20	− 2.52
2	150.20	− 9.42	151.20	− 9.45
3	150.20	− 1.04	150.20	− 0.70
4	150.20	− 4.69	153.20	− 5.86
5	151.20	− 1.85	161.20	− 2.16
6	150.20	− 1.09	150.20	− 0.60
7	155.20	− 4.30	159.20	− 3.24
8	179.20	− 0.30	200.20	− 2.20
9	153.20	− 0.94	156.20	− 0.50
10	224.20	− 1.32	192.20	− 2.65
11	154.20	− 1.21	245.20	− 1.21
12	156.20	− 2.54	151.20	− 1.20

Legend: ms = milliseconds

μV = microvolt

On Table 5 we depict the values related to MMN latency in derivations CZA_1_ and CZA_2_, according to gender.

**Table 5:** Values associated with MMN latency, according to gender and derivation.

Latency (ms)	CZA_1_		CZA_2_	
	F	M	F	M
Minimum	150.2	153.2	150,2	151,2
Maximum	152.2	224.2	161,2	245,2
Mean value	150.7	170.4	153,2	184,0
Standard deviation	0.8367	28.15	4,147	36,16
Variation coefficient	0.56%	16.52%	2,71%	19,65%
P value	0.0011	0.0206		
Result p value	*	*		
	Female		Male	
	CZA	CZA	CZA	CZA
P value (derivation)	0.32	0.39		
Result P value	Ns	Ns		

Legend: * = Significant difference.

Ns = Non-significant difference.

On Table 6 we depict the values related to MMN amplitude in derivations CZA_1_ and CZA_2_, according to gender.

**Table 6:** Values regarding MMN amplitude, according to gender and derivation.

Amplitude (μV)	CZA_1_		CZA_2_	
	F	M	F	M
Maximum	− 9.420	− 4.300	− 9.450	− 3,240
Minimum	− 0.6900	− 0.3000	− 0.6000	− 0,5000
Mean value	− 2.757	− 1.435	− 3.548	− 1,867
Standard deviation	3.326	1.456	3.463	1,013
Variation coefficient	120.65%	101.50%	97.60%	54,25%
P value (gender)	0.1970	0.3496		
P value result	Ns	Ns		
	Female		Male	
	CZA_1_	CZA_2_	CZA_1_	CZA_2_
P value (derivation)	0.42	0.58		
P value result	Ns	Ns		

Legend: Ns = Non-significant difference.

## DISCUSSION

As presented in the literature review, long latency AEP are recorded in values above 80ms (2), between 150 and 380ms (4). In our study, we observed an MMN recording - long latency AEP, considered cognitive between 150 and 250ms (Table 4).

According to Kraus and McGree, 1994 apud4, MMN reaches its ideal value for analysis at school age, therefore, we selected the sample for the present investigation (Table 1 and Figure 2) at an adult age because the goal was to check latency and amplitude characteristics associated with the potential.

MMN is recorded as a negative wave between 150 and 275ms, in a disagreement with the rare stimulus presented together with the frequent stimulus^4^. This was the means to analyzed the recording we used in our study, that is, the rare stimulus wave subtracted from the frequent stimulus wave in derivations CZA_1_ and CZA_2_.

As described in the chapter Materials and Methods (Table 3), the subject under investigation was instructed to watch a video while being stimulated by the frequent and the rare sound, in which the MMN is passively caused, without attention guided to the test^4^.

Kujala et al. (2003)^7^ studied seven subjects with ages between 19 and 23 years. Uther et al. (2003)^12^ studied ten subjects with ages between 18 and 25 years. Winkler et al. (2003)^13^ analyzed three subjects with ages between 18 and 25 years. Näätänen et al. (2004)^19^ analyzed seven subjects with 25 years. The ages of the subjects studied by the aforementioned authors match the ages of the subjects in our study, which varied from 18 years and 2 months to 24 years and 11 months; the females were between 20 years and 5 months and 24 years and 11 months; and from 18 years and 2 months to 23 years and 11 months were males (Table 1 and Figure 2). The number of subjects analyzed in our study, 12 (six females and six males) was larger than the sample of the aforementioned studies.

Sittiprapaporn et al. (2003)^11^ concluded that MMN is more robust when triggered by speech sounds. Pettigrew et al. (2004)^20^ studied the MMN also with vocal stimulus. Such data was not found in our study, because the stimulus we used was tonal.

Giaquinto (2004)^15^ suggested that, in analyzing MMN, the absolute values should not be used for prognosticating alterations, thanks to the large existing variation, without results standardization.

Carrl, Corral and Escera (2005)^23^ studied healthy subjects and observed the presence of MMN in different tonal changes, similar to our study that analyzed healthy subjects (frequent and rare) in different frequencies (1000 and 2000 Hz, respectively).

Näätänen, Jacobsen and Winkler (2005)^25^ suggest that MMN is based on a potential that discriminates any change in intensity, current and precedent. Such data was not analyzed in our study, because the frequent and rare stimuli were equal (75 dBHL).

As we can see on Table 5, the minimum latency value observed in our study was of 150.2ms, for derivations CZA_1_ and CZA_2_; 151.2ms, derivation CZA_2_ and 153.2ms, derivation CZA_1_; the maximum value was 152.2ms and 161.2ms, CZA_1_ and CZA_2_ and 224.2ms and 245.2ms. The mean value was of 150.7ms and 153.2ms derivation CZA_1_ and CZA2; of 170.4ms and 184.0ms, derivation CZA_1_ and CZA_2_ - females and males, respectively.

Variable Amplitude was described on Table 6. The minimum value – 0.69 μV and 0.60 μV, derivation CZA1 and CZA2; maximum value − 9.420 μV and − 9.450 μV, derivation CZA1 and CZA2. The minimum value was −0.30 μV and − 0.50 μV, CZA_1_ and CZA_2_; and the maximum value was −4.300 μV and −3.240 μV, CZA1 and CZA2. The amplitude mean values was −2.757 μV and −3.548 μV, CZA1 and CZA2; of −1.435 μV and −1.867 μV, CZA1 and CZA2 - females and males, respectively.

In the literature we consulted we did not find studies identifying the latency and amplitude values (minimum, maximum, mean and standard deviation) of MMN, for comparison with our findings.

On Table 5 it is possible to see the comparison of variable latency of males and females. The p value (0.001 and 0.0206) suggests a significant difference between the genders, both for derivation CZA_1_, as for derivation CZA_2_. Latency was longer for males in both derivations (mean value of 170.4ms and 184.0ms, CZA_1_ and CZA_2_, respectively).

In the same table, we show the results of the latency variable comparison between right and left sides for males and females. The p value (0.32 and 0.39) indicates that there was no statistically significant difference.

On Table 6 we have a comparison of the variable amplitude for males and females, and there was no significant difference both for derivation CZA_1_, as for derivation CZA_2_ (P = 0.1970 and 0.3496). We also compared the amplitude for the right and left sides, for males and females. P values (0.42 and 0.58) suggest that there are no statistically significant differences.

Although we did not observe statistically significant differences, we identified a potential with greater amplitude for females, for both derivations - CZA_1_ and CZA_2_ (Table 6).

Schirmer, Striano, Friederici (2005)^27^ observed that there is no MMN difference as to gender, in other words, regardless of gender, the event-related potential is an indicator of acoustic change detection. These authors did not indicate that the observation done on the non-difference between genders was related to variable latency or amplitude. If the authors' observation was related to amplitude, it coincides with our findings; however, if their observation is associated with latency, it disagrees from our data, because we registered a statistically significant difference (lower latency for females).

In order to compare derivations CZA_1_ and CZA_2_, we also did not find studies with such data.

## FINAL REMARKS

According to the literature and our findings, we believe there is a need for further studies with MMN, with a larger sample and people at different age ranges in order to standardize data analysis for their use in clinical audiology.

MMN can be used in different clinical conditions with diagnostic purposes, for monitoring and prognosticating the rehabilitation process, after establishing result analysis parameters.

## CONCLUSION

With our findings, we conclude that as far as MMN latency is concerned, the mean value was of 150.7ms (CZA_1_) and 153,2ms (CZA_2_), for females; of 170.4ms (CZA_1_) and 184.0ms (CZA_2_), for males. There was a statistically significant difference between males and females for derivations CZA_1_ and CZA_2_ (P=0.0011 and 0.0206). Lower latency for females and higher for males. There was no statistically significant difference between the right and left sides for both genders (P = 0.32 and 0.39).

As to MMN amplitude, the mean value was of − 2.757μV (CZA_1_) and −3,548μV (CZA_2_), for females; and of −1.435μV (CZA_1_) and −1.867μV (CZA_2_), for males. There was no statistically significant difference between males and females for derivations CZA_1_ and CZA_2_ (P = 0.1970 and 0.3496) nor for the right and left sides, for both genders (P = 0.42 and 0.58) larger amplitude for females and lower for males.
